# LETTER TO THE EDITOR Abnormal Responses of Keloid Tissue to Wounding Identified Using In Vitro Model System

**Published:** 2012-04-12

**Authors:** Dorothy M. Supp, Kathryn Glaser, Jennifer M. Hahn, Kevin L. McFarland, Steven T. Boyce

**Affiliations:** ^a^The Research Department, The Shriners Hospitals for Children—Cincinnati; ^b^Department of Surgery, The University of Cincinnati, College of Medicine, Cincinnati, OH

Dear Sir,

Keloids are thick, raised scars that represent an extreme form of abnormal scarring. Unlike normal scars, keloids extend beyond the original wound margin and rarely regress; instead, they tend to proliferate indefinitely.^1^^-^4 These bulky scars can significantly impair function due to itching, pain, and decreased range of motion^5^ and can negatively impact psychosocial well-being and overall quality of life.^5^^-^7 Although many different therapeutic modalities exist, keloids are extremely resistant to treatment and have a high rate of recurrence.^1^^,^^2^^,^^8^^-^10 Development of effective, targeted interventions has been limited due to an incomplete understanding of the pathophysiology of keloid scarring. Furthermore, because keloid scarring is only found in humans, there are no animal models of keloid scarring, which has hindered the evaluation of novel therapies. Ethical considerations preclude wound healing studies in patients susceptible to keloid scarring. Therefore, in the absence of suitable animal models, organotypic models represent a feasible alternative for investigation of wounding in keloid tissue. In the current study, we investigated engineered skin substitutes (ESS) composed of keratinocytes, fibroblasts, and collagen-glycosaminoglycan biopolymers, as an in vitro organotypic keloid model to analyze changes in gene expression in response to wounding.

Primary human fibroblasts and keratinocytes were isolated and cultured^11^^,^^12^ from excised keloid scar or normal skin, collected with University of Cincinnati Institutional Review Board approval. ESS were prepared by sequential inoculation of fibroblasts and keratinocytes onto approximately 40 cm^2^ collagen-glycosaminoglycan biopolymer sponges.^11^^,^^13^^,^^14^ After 7 days of culture at the air-liquid interface, ESS were cut in half, and one piece of each was wounded using a skin graft mesher; approximation of the cut edges permitted healing of the wounded ESS to occur.

Histological analysis of sections of normal and keloid ESS 14 days after keratinocyte inoculation demonstrated a well-stratified epidermal layer and a dermal component populated with fibroblasts (Figs 1a-1f). Seven days after wounding, migration of keratinocytes into the wound edge occurred to varying degrees in both normal and wounded ESS. In keloid ESS, newly synthesized collagen was visible in the area adjacent to the wound, as evidenced qualitatively in trichrome-stained sections. This was not observed in the normal ESS after wounding.

Immunohistochemistry was performed to localize periostin, a matricellular protein that has been shown to be expressed at higher levels in keloid fibroblasts than in normal fibroblasts.^15^ In contrast to normal ESS or nonwounded keloid ESS, high levels of periostin were detected in wounded keloid ESS and were localized to the upper dermis and dermal-epidermal junction in the region of the healing wound (Figs 1g and 1h).

Quantitative real-time PCR^16^^,^^17^ was used to analyze expression of genes previously implicated in keloid scarring: type 1 collagen pro-alpha 1 and 2 chain genes (COL1A1 and COL1A2); matrix metalloproteinases 1 and 3 (MMP1 and MMP3); and periostin (POSTN)^15^^,^^18^^-^22 (Fig 2). Expression levels for COL1A1, COL1A2, and POSTN were higher in nonwounded keloid ESS than in nonwounded normal ESS. Upon in vitro wounding, expression of these genes was slightly elevated in normal ESS but the differences were not statistically significant. In contrast, COL1A1, COL1A2, and POSTN expression levels were significantly increased in wounded keloid ESS. MMP1 and MMP3 were expressed at lower levels in keloid ESS compared with normal ESS and were not significantly increased after wounding.

The results obtained using ESS as an in vitro organotypic model are consistent with the hypothesis that an imbalance of extracellular matrix (ECM) production and degradation after wounding leads to keloid scarring. In our organotypic model, keloid cells respond to injury with an exaggerated increase in ECM production. The expression of genes involved in ECM breakdown is insufficient to counteract this significant increase in matrix production. We propose that this in vitro model will provide a useful system for further identification of abnormal interactions between keloid fibroblasts, keratinocytes, and ECM, and screening of novel therapies.

## Figures and Tables

**Figure 1:**
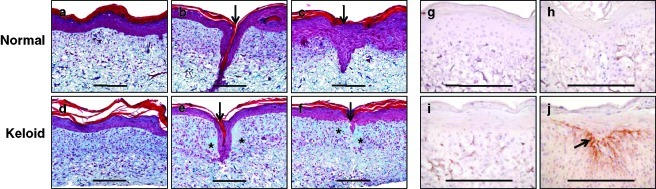
Histological analysis of normal and keloid ESS. (*a-f*), Representative formalin-fixed, paraffin-embedded sections of ESS from day 14 of in vitro incubation, stained with Masson's trichrome. (*a*) Nonwounded normal ESS. (*b-c*), Wounded normal ESS. (*d*), Nonwounded keloid ESS. (*e-f*) Wounded keloid ESS. In these sections, the epidermal layer stains dark reddish-pink; cell nuclei stain reddish-pink to reddish-blue; the reticulations of bovine collagen from the biopolymer sponge material stain dark blue; and the newly synthesized human collagen stains bright greenish-blue. Locations of “healing” wounds in *b*, *c*, *e*, *f* indicated by arrows. Note the increased collagen deposition in the wounded keloid ESS (asterisks) compared with control unwounded or normal wounded ESS. (*g*-*j*) Immunohistochemical localization of periostin, a matricellular protein involved in collagen fibril formation, in frozen sections of normal (*g*-*h*) and keloid (*i*-*j*) ESS. The levels of periostin were below detection in control non-wounded normal (*g*) or wounded normal ESS (*h*), and were low in nonwounded keloid ESS (*i*). However, periostin signal was readily detected in keloid ESS after wounding (*j*) and was localized to the upper dermis and dermal-epidermal junction in the region of the healing wound (brown staining; arrow). Immunohistochemical staining of control sections, without primary antibody, showed no positive signal (data not shown). Scale bars = 200 µm in all sections.

**Figure 2:**
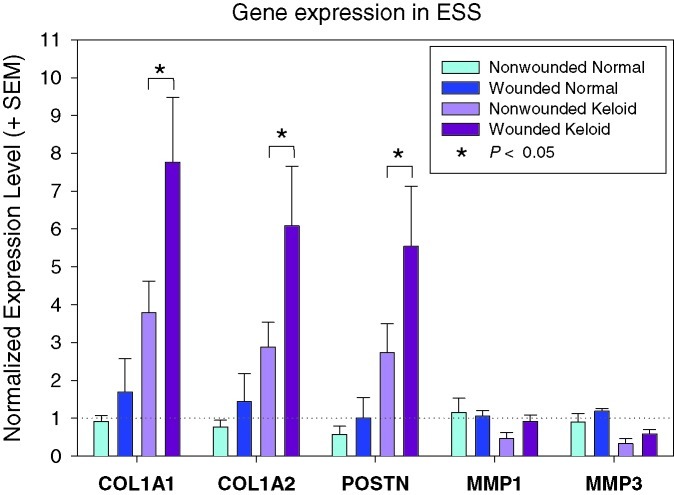
Expression of COL1A1, COL1A2, POSTN, MMP1, and MMP3 mRNA was quantified using real time PCR. Expression levels were normalized to the level for one nonwounded normal ESS sample, and mean normalized levels + standard error of the mean are plotted. Expression of COL1A1, COL1A2, and POSTN was higher in nonwounded keloid ESS compared with normal ESS. Upon in vitro wounding, no significant difference was observed in normal ESS, but COL1A1, COL1A2, and POSTN expression levels were significantly increased in wounded keloid ESS (*P < 0.05). MMP1 and MMP3 were expressed at lower levels in keloid ESS compared with normal ESS, and were increased in keloid ESS after wounding, but the differences were not statistically significant. The mean MMP1 and MMP3 levels in wounded keloid ESS were lower than in nonwounded or wounded normal ESS. Statistical analysis was performed using repeated measures analysis of variance.
